# Association between aortic stiffness measured by cardiovascular magnetic resonance and sub-clinical carotid atherosclerosis in young adults

**DOI:** 10.1186/1532-429X-11-S1-O43

**Published:** 2009-01-28

**Authors:** Ilias Kylintireas, Colin Cunnington, Corinne Trevitt, Jonathan Diesch, Stefan Neubauer, Matthew Robson, Paul Leeson

**Affiliations:** University Of Oxford, Oxford, UK

**Keywords:** Internal Carotid Artery, Cardiovascular Magnetic Resonance, Arterial Stiffness, Pulse Wave Velocity, Aortic Stiffness

## Introduction

Increased arterial stiffness is associated with increased cardiovascular risk in later life. Cardiovascular magnetic resonance (CMR) allows direct assessment of arterial stiffness by imaging the elastic properties of the aorta. This provides measures of both global (pulse wave velocity, PWV) and regional (aortic distensibility) aortic stiffness. Assessment can be combined with precise measurement of carotid atheroma burden, a marker of early sub-clinical atherosclerosis. We investigated whether aortic stiffness in early adult life is already associated with early changes in carotid structure.

## Purpose

To determine whether aortic stiffness – quantified by cardiovascular magnetic resonance as pulse wave velocity (PWV) and aortic distensibility (AD) – is associated with early atherosclerosis-related structural changes in young adult life.

## Methods

Thirty young healthy volunteers (aged 23–33) (without history of cardiovascular disease or classical risk factors for atherosclerosis) underwent CMR for measurement of aortic function and carotid wall imaging.

Aortic distensibility was measured from breath-hold ECG-gated, steady state free precession (SSFP) images. Distensibility was calculated as the relative change in area divided by the central pulse pressure. Pulse wave velocity was measured from an ECG-gated, free breathing, spoiled gradient echo phase-encoded acquisition. The transit time method was used for the calculation of pulse wave velocity (PWV). T1 weighted black blood turbo spin echo (TSE) cross-sectional images of both carotid arteries, centred at the lowest bifurcation were used for atheroma burden measurements (plaque index represented cross-sectional vessel wall area/total cross-sectional vascular area). Plaque index was averaged for the common carotid (CPI), the carotid bulb (BPI) and the internal carotid artery (IPI).

## Results

**Figure 1:**
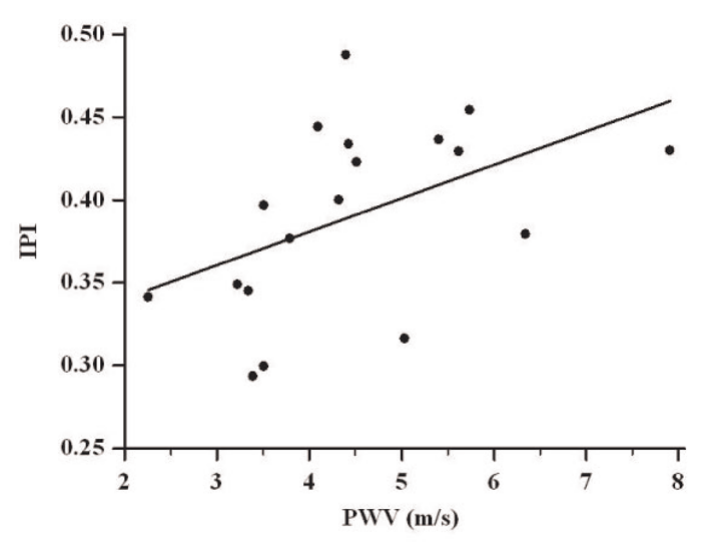
Figure 1

CMR-derived PWV over the whole length of the aorta was correlated with carotid plaque index (r = 0.480, P < 0.05) (Figure 1.) particularly of the internal carotid artery. Regional measures of aortic distensibility and pulse wave velocity were unrelated to carotid atheroma burden. Applying a multiple regression analysis model (including applicable risk factors, demographics and anthropometric measurements) PWV was the sole independent predictor of IPI [β = 0.02(± 0.009), P < 0.05, R^2^ = 0.23].

## Conclusion

Aortic stiffness assessed by CMR is associated with early atherosclerosis-related changes in carotid arteries in young adults.

